# Emergence of Antibiotic-Producing Microorganisms in Residential Versus Recreational Microenvironments

**DOI:** 10.9734/BMRJ/2013/3205

**Published:** 2013-04-23

**Authors:** Yvon Woappi, Prashant Gabani, Om V. Singh

**Affiliations:** 1 Division of Biological and Health Sciences, University of Pittsburgh, Bradford, PA-16701, USA

**Keywords:** 16S rRNA, antibiotics, microorganisms, antibiotic potency, zone of inhibition, pathogens, antibiotic resistance

## Abstract

**Aims:**

To identify novel antibiotic-producing microbial strains with unprecedented pertinence. We hypothesize that site-specific soil samples will contain a variety of antibiotic-producing species (APS) with diverse specificity of molecular elements.

**Place and Duration of Study:**

Laboratory of Microbiology, Division of Biological and Health Sciences, University of Pittsburgh, Bradford, PA-16701, USA, between August 2010 and May 2011.

**Methodology:**

The environmental soil samples were collected from residential and recreational sites in Southern, PA, USA at longitude: −76 42 21.7116, latitude: 39 56 35.7252; approximately 201 meters above sea level. Over 70 natural antibiotic-producing soil bacteria were screened against 19 pathogenic microorganisms. Agar-plug assay was established to identify the antibiotics’ potency and pathogenic inhibitory index calculations were employed to measure the inhibitory potential of each isolate; 16S rRNA sequencing was used for microbial classification.

**Results:**

A total of 71 microorganisms from residential soil demonstrated zones of inhibition (ZOI), followed by 9 organisms from recreational soil sample. A total of 15 bioactive strains demonstrated convincing growth inhibitory properties against 16 clinically relevant pathogens; 40% revealed pDNA presence, of which 67% exhibited stringent potencies against *S. aureus*. We observed a highly bioactive residential soil microbiota compared to recreational soil.

**Conclusion:**

16S rRNA sequence analysis corroborated several of the species belonging to *Enterobacteriaceae*, *Xanthomonadaceae*, and *Bacillaceae*. These findings may indicate a co-evolutionary biosynthesis of novel antibiotics driven by the increase of bioactive microbiota in residential environments.

## 1. INTRODUCTION

The microbial products of secondary metabolism carry an important role in human health, providing roadmaps for the biosynthesis of many synthetic and semi-synthetic drugs. In nature, microbial bioactive products are present as mycotoxins or bacteriosins derived from filamentous and non-filamentous bacteria and fungi. The soil-based actinomycetes have been the source of countless drugs, from streptomycin and actinomycin, to erythromycin and vancomycin [1]. Natural soil harbors over 109 microorganisms/ gram and provides an ideal reservoir for bioactive microbiota, which springs virtually all clinical antibiotics used today [2,3]. Today nearly 500 antibiotics are found each year and over 80% of antibiotics in clinical use are obtained from soil isolates [1]. These bioactive microorganisms are most abundantly present at the top few inches of the soil, in soil containing straw and agricultural products [2,4]. Studies have also suggested that soil from areas containing residential-derived materials, such as human fecal matter, contains 10–20 times more antibiotic resistant strains than recreational or industrial soils [5]. Consequently and in addition to the elevated use of therapeutic drugs in urban or residential environments, residential soils contain exceptionally high bioactivity and are abundant reservoirs of antibiotic-resistant microorganisms[5,6].

Plasmid-mediated antibiotic producing genes can be acquired environmentally by transposition from one microorganism to another [2]. The phenomenon of intracellular plasmid acquisition enables antibiotic-producing bacteria to co-exist with antibiotic-resistant strains through the synthesis of novel bioactive compounds, which allows them to combat their continuously evolving antibiotic resistant counterparts [7,8]. This sophisticated co-evolutionary adaptation is perpetual within microbial-rich soil and represents a great reservoir for novel, natural (non-synthesized) antibiotics. The successful advancement of non-synthetic and synthetic antibiotic therapeutic applications therefore requires constant identification and characterization of natural antibiotic-producing microorganisms.

Due to their topographical inconvenience, little research has been thoroughly conducted on residential microenvironments. We aimed to explore the natural antibiotic biosynthesis potentials of microorganisms isolated from soil collected at residential and recreational sites and hypothesized that site-specific soil samples will contain a diversity of antibiotic-producing strains (APS) with a variety of molecular elements. The molecular characterization of several isolated growth-inhibitory species revealed plasmid DNA (pDNA) presence. We observed a higher antibiotic-producing community in residential soil compared to recreational soil. The diverse microbial population, approximately 105 microorganisms per gram of soil, displayed copious amounts of soil-dwelling APS against 16 clinically relevant pathogens; 16S rRNA sequencing revealed unique heterogeneity traits in these APS.

## 2. MATERIALS AND METHODS

### 2.1 Strains Isolation

Environmental soil samples were collected in Southern, PA, USA from 2010–2011 at longitude: −76 42 21.7116, latitude: 39 56 35.7252, approximately 201 meters above sea level. Two primary locations were selected for sample collection: a recreational area with high human traffic (i.e. 94 yard of an industrial site) and an authorized residential property with an existent human populace. Collected samples were placed in polyethylene bags and stored in dark at 4 °C until analyzed for antimicrobial properties. The soil type obtained at both locations was MT. Airy-Glenelg-Linganore soil (PA087; soilmap.psu.edu).

Due to the heterogeneous nature of soil samples, three different growth medium, Tryptic Soy Agar (TSA), Potato Dextrose Agar (PDA), and Nutrient Agar (NA), were utilized for the emergence and isolation of microorganisms. The antibiotic-producing abilities of the collected microbiota was tested in triplicates and assayed by growth inhibition zone against 19 clinically relevant pathogenic microorganisms obtained from American Type Culture Collection (ATCC) *: Streptococcus pneumoniae, Staphylococcus aureus, Streptococcus thermophilus, Listeria monocytogenes, Ochrobae anthropic, Staphylococcus sciuri, Kocuria kristinae, Klebsiella pneumoniae, Escherichia coli, Enterococcus saccharolyticus, Klebsiella oxytoca, Streptococcus zooepidemicus, Candida albicans, Enterococcus faecalis, Serratia marcescens, Saccharomyces cerevisiae, Micrococcus luteus, Bacillus cereus,* and *Pseudomonas fluorescens*.

### 2.2 Primary and Secondary Screening

All pathogenic microorganisms were spread on 90-mm polystyrene petri dishes filled with TSA, PDA, and NA medium, respectively. The collected bioactive soil samples (1–5g) from each 112 location were dried at 37°C and sprinkled (~ 80–100 particles) on dishes spread with each respective ATCC pathogen. Control plates were merely spread with ATCC pathogens and incubated at 37°C. After 48 h incubation period, microorganisms displaying clear zones of inhibition against the pathogens were sequestered onto plates of respective medium and assigned numbers. Repeated streaking purified the isolated microorganisms and single-cell colonies were picked up for further screening and temporary storage at 4°C in medium-agar-filled slants. Microorganisms displaying little or no pathogenic inhibitory properties were retested and discarded if scarce antibiotic biosynthesis persisted. Purified microorganisms obtained from this primary screening were further tested for growth-inhibitory properties by using the perpendicular cross-plate method (PCPM) [9] against all 19 pathogenic microorganisms. Antibiotic-producing isolates were considered as any microorganisms which inhibited the growth of another microbe within its perimeters [10]. Plates were incubated at 37°C and analyzed after regular time intervals (12 h, 16 h, 24 h, and 32h) for zones of inhibition (ZOI) across the junctions. Microorganisms displaying little or no growth inhibitory properties were retested and discarded if insufficient antibiotic biosynthesis persisted. APS revealing inhibitory zones against one or more pathogen were further purified and maintained on agar slants at 4°C in respective growth medium. The APS were grown in an orbital shaker incubator at 37°C overnight in respective liquid culture medium and rescreened against all 19 ATCC microorganisms using PCPM as aforementioned.

#### 2.1.1 Antibiotic production assay

Due to their potential industrial significance, we aimed to thoroughly investigate antibiotic secreting abilities among the screened APSs. Secondarily screened APSs and blank controls were grown in 50 ml of respective liquid growth medium (TSB, PDB, and NB) at 37°C, 120 rpm for 24 h. Cells were pelleted (OD600 2.2–2.5) by centrifugation at 22°C, 10,000g for 10 min. The supernatant was collected and evaporated by sterile airflow incubation at 37°C. Desiccated supernatant was sterilized and reconstituted in 1 ml respective growth medium and stored at −20°C until analyzed for antibiotic properties through agar-plug assay.

### 2.3 Agar-Plug Assay

Agar-plug assay was adopted from Bechard et al. [11] with a 5-mm sterile cork borer utilized to plug wells on respective agar plates (NA, PDA, and TSA) lawned with pathogens. Each well was filled with 20 μL of reconstituted supernatant, respectively. In parallel, blank growth medium with no organism was evaporated, reconstituted, and loaded in a well within each respective plate. The latter were incubated at 37°C and examined for ZOI, a clear halo around the wells, after regular time intervals (12 h, 16 h, 24 h, and 32 h). Isolates displaying remarkable inhibitory properties were kept for further analysis. APS with little inhibitory properties were retested at 30 μL and 40 μL per well and discarded if insufficient antibiotic biosynthesis persisted. We utilized photographic pixel quantification (Adobe Photoshop CS6) to distinguish which isolates developed the most unpenetrated inhibitory perimeters [12]. The difference in pixel density between the streaked plates and its ZOI area was calculated and used to assign an inhibitory potency value ranging from 0–5 to each isolate; the sum of these values determined the Antibiotic Potency (A.P). The latter, along with ZOI diameter measurements and the range of pathogenic inhibition (i.e. number of pathogens inhibited), was used to calculate the Pathogenic Inhibitory Index (P.I.I) of each isolate, providing a holistic view isolates’ growth inhibition potentials:

$$\frac{1}{3}\sum_{i=1}^{3}\mu P.I.I+\mu A.P+\mu \mathrm{Inhibitory}\;\mathrm{Range}$$

### 2.4 Plasmid DNA Extraction

The screened APSs were characterized for the presence of pDNA. pDNA was isolated from overnight grown APSs (OD600 2.2–2.5) using PureLink^™^ Quick Plasmid Miniprep kit (Invitrogen, USA). The isolated pDNA was visualized on 1% ( w/v) agarose gel electrophoresis stained with ethidium bromide.

### 2.5 Phylogenetic Analysis

Cellular DNA from three APSs: Y14p, Y16 and Y40p, which shared analogous morphologies and growth inhibitory properties as most soil isolates, was extracted using PureLink^™^ Genomic DNA MiniKit K1820-01 (Invitrogen, USA). The 16S rRNA gene sequences from these three APSs were then amplified using universal primer (F-518: CCAGCAGCCGCGGTAATACG, R-800: TACCAGGGTATCTAATCC) and sequenced at Macrogen Service Center (Rockville, MD, USA). All sequences were compared with closest relatives from GenBank and Ribosomal Database Project ( RDP) release 10 (http://rdp.cme.msu.edu/index.jsp). Phylogenetic trees were constructed by neighbor-joining method (NJ) with pairwise deletion of gaps in RDP database.

#### 2.5.1 Nucleotide sequence accession numbers

The sequences of all pure cultures have been deposited in GenBank database under accession numbers JQ956432, JQ956433, and JX121858 for Y14, Y16, and Y40, respectively.

### 2.6 Statistical Analysis

All experiments were carried out in triplicate, and the experimental results represent the mean of three identical sets of experiments. One-way ANOVAs followed by least significant difference (Tukey’s honestly significant difference) were performed to evaluate the potential significant differences.

## 3. RESULTS AND DISCUSSION

### 3.1 Isolation of Antibiotic-Producing Microorganisms

The primary screening of residential and recreational soils yielded a total of 71 and 9 promising APS with growth inhibitory properties, respectively (Table 1). On NA medium, 52 (73.2%) isolates from residential soil displayed growth inhibitory zones against 19 pathogenic microorganisms, while all nine (100.0%) isolates from recreational soil displayed inhibitory properties (Table 1). A total of 12 (16.9%) isolates from residential soil showed inhibitory zones on PDA plates, but only seven (9.9%) demonstrated inhibitory zones on TSA medium (Table 1).

All isolates selected from primary screening were re-screened by PCPM. Most isolates obtained from recreational sites showed insufficient inhibitory capacities against all nineteen pathogenic microorganisms during secondary screening. Only one isolate (L59) from recreational sites insistently exhibited growth inhibitory properties, inhibiting *B. cereus*, *C. albicans*, *P. fluorescence*, *S. aureus*, and *S. zooepidemicus* on NA medium (Table 2). The broad range of isolates from residential soil samples, i.e. 11 (21.2%), 4 (33.3%), and 4 (57.5%) isolates, displayed sufficient growth inhibitory properties on NA, PDA, and TSA media, respectively (Table 1). Recreational soil did not reveal isolates with growth inhibitory properties on TSA or PDA medium, and overall yielded nearly six times less APS than residential soil (Table 1, *P = .05*).

To determine the potential extracellular production of antibiotics from isolates screened by PCPM, agar plug assay was used. A total of eight (88.89%; i.e. Y40p, Y17p, Y29p, Y16, Y16a, Y39, Y44, and Y49), four (100.0%; i.e. Y63p, Y67p, Y64a, and Y64b) and two (100%; i.e. Y13 and Y67p) isolates with growth inhibitory zone formations on NA, TSA, and PDA media respectively revealed extracellular antibiotic production abilities (Table 1). The single remaining recreational soil-derived isolate (L59) persisted through this screening. Overall, a total of 15 different antibiotic-producing isolates with sufficient inhibitory potential were isolated and further characterized. The single remaining recreational soil-derived isolate (L59)persisted through this screening. Overall, a total of 15 different antibiotic-producing organisms with sufficient inhibitory potential were isolated and further characterized. During our study, isolates’ inhibitory capacities alternated at different screening stages. At some stages they displayed growth suppressive abilities towards certain pathogens but lost or switched these inhibitory abilities toward other pathogens during a different screening stage (Table 1–2). These observations may indicate the presence of highly spontaneous antibiotic-specificity swapping involved interdependently in antibiotic synthesis between soil isolates within an optimal microenvironment, and support previous studies which specify the robust influence of neighboring microorganisms on surrounding bacterial antibiotic secretion [13].

### 3.2 Growth-Inhibitory Assessment

Individual values of average zone of inhibition (ZOI) of each isolate are summarized in Table 3, and reveal Y14 with the largest ZOI (10.16 mm), demonstrated against pathogen *K. kristinae* on PDA media. Isolate Y13 exhibited its maximum inhibitory perimeter against *S. marcescess* (9.40 mm). Y14p and Y44 displayed their maximum inhibitory perimeters against *K. kristinae* (10.16 mm and 2.01 mm, respectively). Y16 and Y67p displayed their largest inhibitory perimeters against *L. monocytogenes* (2.54 mm and 2.54 mm). Y16a revealed maximum ZOI against *O. anthropi* (2.54 mm). The isolate Y17p exhibited its maximum inhibitory perimeter against *P. fluorescence, C. albicans* and *S. aureus* (2.54 mm, 2.54 mm, and 2.54 mm). Y29p displayed its largest inhibitory zone against *P. fluorescence* and *S. aureus* (2.54 mm and 2.54 mm). Similarly, Y39 had its greatest inhibitory perimeter against *En. saccharolyticus* (2.29 mm). Y40p greatest inhibitory perimeter was against *P. fluorescence* (2.54 mm). The isolates Y49 and L59 maximum inhibitory perimeter was against *S. aureus* (6.35 mm and 6.10 mm). Y63p largest ZOI was against *S. zooepiderimicus* (3.81 mm). Y64a maximum ZOI was against *C. albicans* (2.54 mm). Y64b largest ZOI was against *L. monocytogenes* (5.08 mm).

**Fig. 1:**
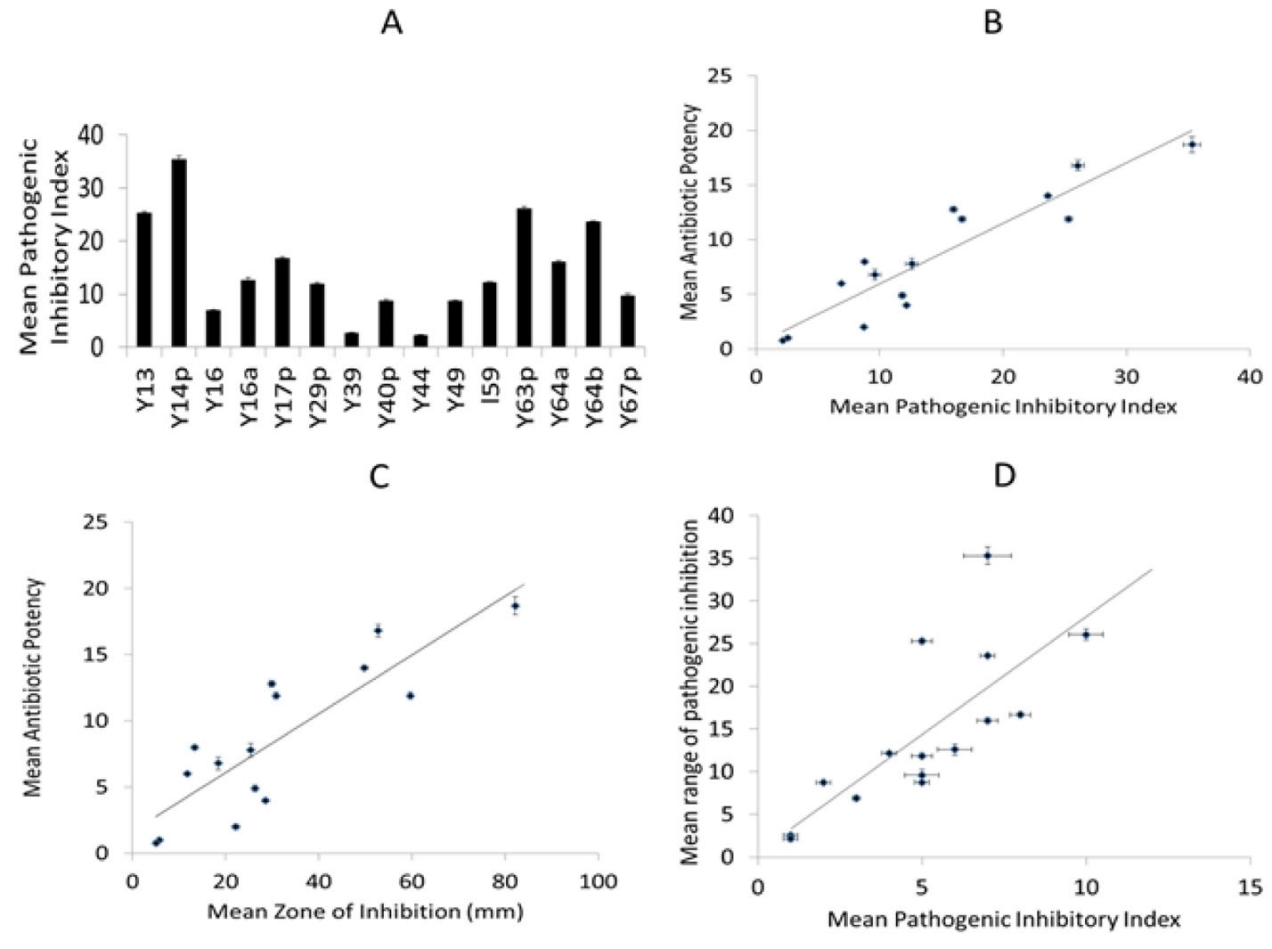
Mean pathogenic inhibitory index of soil isolates A: Several soil isolates revealed elevated mean pathogenic index. B: Positive correlation displayed between APS’ antibiotic potency and Pathogenic Inhibitory Index (Correlation coefficient=0.96, P = .05). Mean (± S.E.M). C: Positive correlation demonstrated between antibiotic potency and zone of inhibition measurement (c.c=0.84, p> 0.05). Mean (± S.E.M). D: Positive correlation displayed between the range of pathogenic inhibition and the Pathogenic Inhibitory Index (c.c=−0.20, * P = .05)

Furthermore, the APSs antibiotic potency (A.P) and average range of growth inhibition of pathogenic microorganisms were determined. Isolates Y17p and Y14p demonstrated the greatest A.P, which was observed against *P. fluorescence* (A.P. = 5) and *M. luteus* (A.P. = 5), respectively. Y63p however had the widest range of pathogenic inhibition, revealing growth suppressive capacities against more than 60% of the screened clinically-relevant pathogens *L. monocytogenes, En. saccharolyticus, K. kristinae, K. faecalis, M. luteus, O. anthropi, S. cerevisiae, S. aureus, S. scuiri, and S. zooepiderimicus*. Among the isolated microorganisms, isolates Y14p, Y63p, and Y13 had the greatest P.I.I (*i.e.* 35.3, 26.1, and 25.3), respectively, as the combination of their range of pathogenic inhibition, A.P, and ZOI was the greatest (Fig. 1a). Isolates Y44, and Y39 demonstrated the weakest A.P, respectively. No isolate was able to inhibit *K. pneumoniae*, *K. oxytoca*, and *S. thermophilus*. As anticipated, positive correlations existed between A.P, P.I.I (c.c=0.85,*P = .05*), ZOI (c.c=0.92, *P = .05*), and the range of pathogens inhibition (Fig. 1d; c.c= 0.76; *P = .05*) (Fig. 1b, c).

### 3.3 Detection of Plasmid DNA

**Fig. 2:**
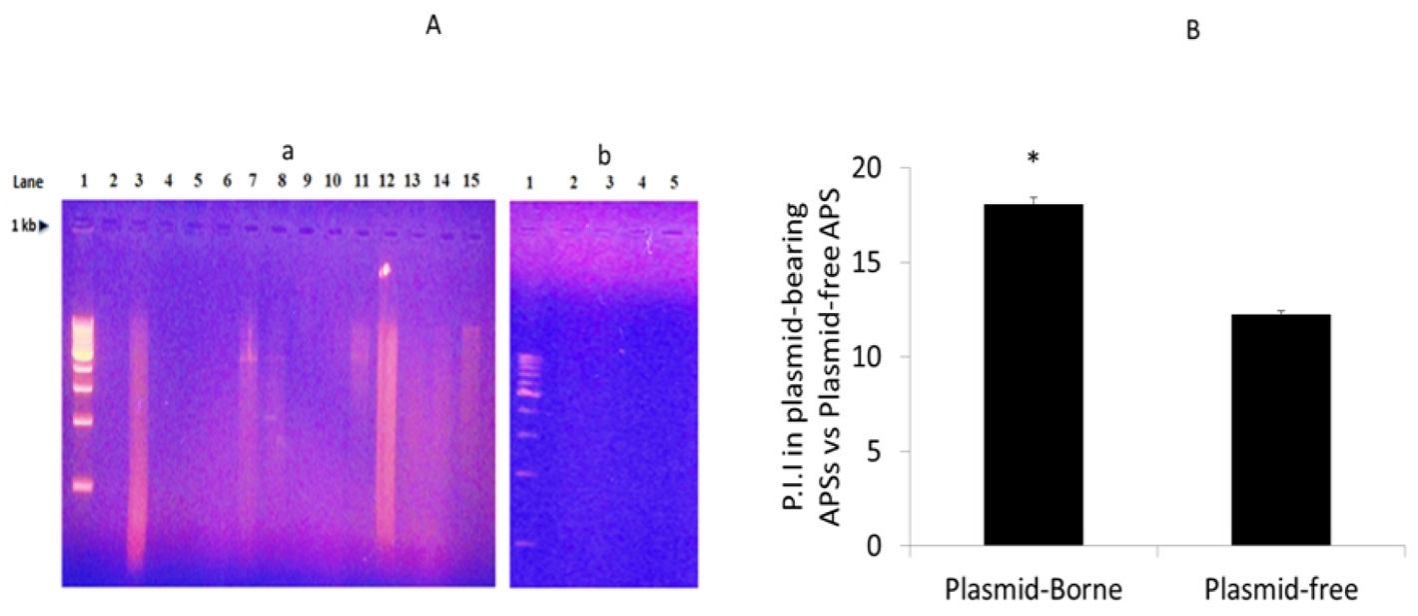
Fig. 2A. Plasmid DNA profiles and pathogenic Inhibitory index of soil-isolated antibiotic-producing microorganisms A: (a) Isolates Y14, Y17, Y29, Y40, Y63, and Y67 (Lanes #3, 7, 8, 11, 12, and 15, respectively) revealed pDNA presence, while isolates 16a, 14, 16, 17 (lanes 2,3,4,5 respectively) did not; (b) Isolates Y59, Y63, Y13, Y64a, Y64b, Y67 (lanes 11, 12, 13, 14, 15, respectively) did not possess pDNA. 1 kb ladder was used. B: Mean Pathogenic Inhibitory Index of pDNA-bearing and pDNA free soil isolates (*P = .05) Mean (± S.E.M)

To determine the molecular elements which may be involved in antibiotic production in site-specific soil isolates, we looked for plasmid DNA existence and found it in several residential soil-derived APSs, designated as Y14p, Y29p, Y40p, Y17p, Y63p, and Y67p (Fig. 2a). These isolates exhibited significantly greater P.I.I than plasmid-free APSs (Fig. 2b). Four plasmid bearing APSs (Y14p, Y17p, Y63p, and Y67p) demonstrated substantial growth suppression against *L. monocytogenes* and *S. aureus*, but the broadest inhibition was demonstrated against *P. fluorescens* by 83.3 % of *p*DNA-bearing APSs (Table 3). Contrarily, only 22.2% of *p*DNA free isolates (Y13, Y16a, and L59) were able to inhibit *P. fluorescence*. Some *p*DNA-free isolates (*i.e.* Y44, Y13, Y64b, Y16, Y16a, Y64a), however displayed strong inhibitory properties against either *S. sciuri,* or *S. cerevisiae*, but the majority (55.6%; i.e. Y44, Y16, Y16a, Y49, Y64a) demonstrated inhibition against *K. kristinae* (Table 3).

The ability of residential soil-dwelling *p*DNA isolates to inhibit both gram-negative and positive bacteria suggests they possess neutrophilic, as well as aciduric and chromate-like properties, producing antibiotics similar to β-lactam, 6-anilinouracils (AUs), tetracyclic, oxytertracycline or other polycyclic compounds which certainly present potential for food-borne and nosocomial disease applications [17]. Generally, *p*DNA-free APSs demonstrated potent antibiotic properties against *S. sciuri* and *K. kristinae,* gram-positive facultative anaerobes which are normal inhabitants of human tissue and have been cited to cause a wide range of infectious diseases [18]. Among the screened pathogens was *S. aureus*, the pathogen responsible for Methicillin-Resistant *Staphulococcus aureus* (MRSA) development, which has been identified as the most common cause of nosocomial diseases, often acquired outside the household and brought back into residential communities by returning patients [19]. In our studies, over 66.6% of residential plasmid-borne isolates conferred inhibitory qualities against *S. aureus* (Table 3), indicating that residential soil could be a substantially evolving reservoir for antibiotic-producing isolates, constantly adapting to pathogens obtained from various environments and subsequently developing novel and specific antibiotics against resistant strains.

### 3.4 Phylogenetic Relationships of Potent Antibiotic-Producing Microorganisms

**Fig. 3:**
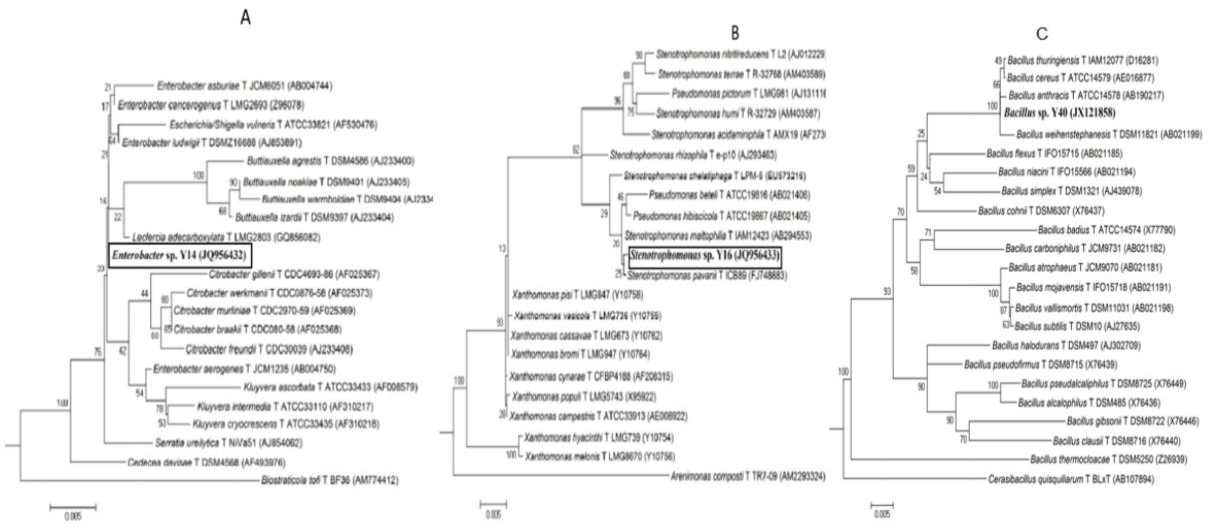
16S rRNA gene sequence based neighbor-joining phylogenetic tree showing genomic relationships of isolates Y16, Y14 and Y40 16S rRNA gene sequence based neighbor-joining phylogenetic tree showing relationship between Y14 **(a)**, Y16 **(b)**, and Y40 **(c)** and related microbial strains. Y14, Y16 and Y40 showed close proximity with the genus Enterobacter sp., Stentrophomonas sp., and Bacillus sp., respectively. Microorganisms Biostraticola tofi T BF36, Sinobacter flavus T CWKD4, and Cerasibacillus quisquiliarum T BLxT were used as out-groups for Y14, Y16, and Y40, respectively. Numbers at the nodes indicate levels of bootstrap support based on neighbor-joining analysis of 1000 resampled datasets. GenBank accession numbers are given in parenthesis. Bar, 5 substitution for 1000 nucleotide positions.

Due to their foremost ZOI perimeters and their consistency in pathogenic inhibition, the three soil isolates Y14p, Y16, and Y40p were attempted to identify using 16S rRNA based sequence analysis of phylogenetic relationship. The partial sequences of 956 bp, 958 bp, and 953 bp of Y14p, Y16, and Y40, respectively, were compared with closest relatives from GenBank and Ribosomal Database Project ( RDP) release 10 (http://rdp.cme.msu.edu/index.jsp). The sequence analysis of 16S rRNA revealed the isolates Y14p, Y16, and Y40 to be closely related to family *Enterobacteriaceae*, *Xanthomonadaceae*, and *Bacillaceae*, respectively (Fig. 3a–c). All three organisms (i.e. Y14p, Y16, and Y40) showed close proximity with genus *Enterobacter s*p. *Stenotrophomonas sp*., and *Bacillus sp*., respectively (Fig. 3a–c). Further, the sequences for Y14p, Y16, and Y40 were deposited in GenBank database under the accession numbers JQ956432, JQ956433, and JX121858, respectively. The GenBank and RDP profiles of isolate Y16 closely matched that of *Stenotrophomonas* sp., gram-negative obligate aerobes found in water and soil. They are cited as pDNA carriers and have been noted for their robust resistance to antibiotics as well as their role in nosocomial infections [21]. They produce xanthobaccins, with strict antimycological properties, lacking the ability to inhibit gram-positive or gram-negative bacteria [22]. As expected, isolate Y16 inhibited *C. alibicans or S. cerevisia* and did not exhibit inhibitory properties against Gram-negative pathogens. It did however demonstrate a strong growth suppressive ability against Gram-positive pathogens. Conversely, the genomic profiles of isolate Y14p closely resembled that of *E. ludwiggi* which has been cited as a potential pathogen to humans [21,23]. In our findings, Y14p displayed a wide-range of inhibitory abilities against gram-positive, gram-negative, and mycological pathogens.

### 3.5 Paradigm of Antibiotic-Producing Microbiota

**Fig. 4:**
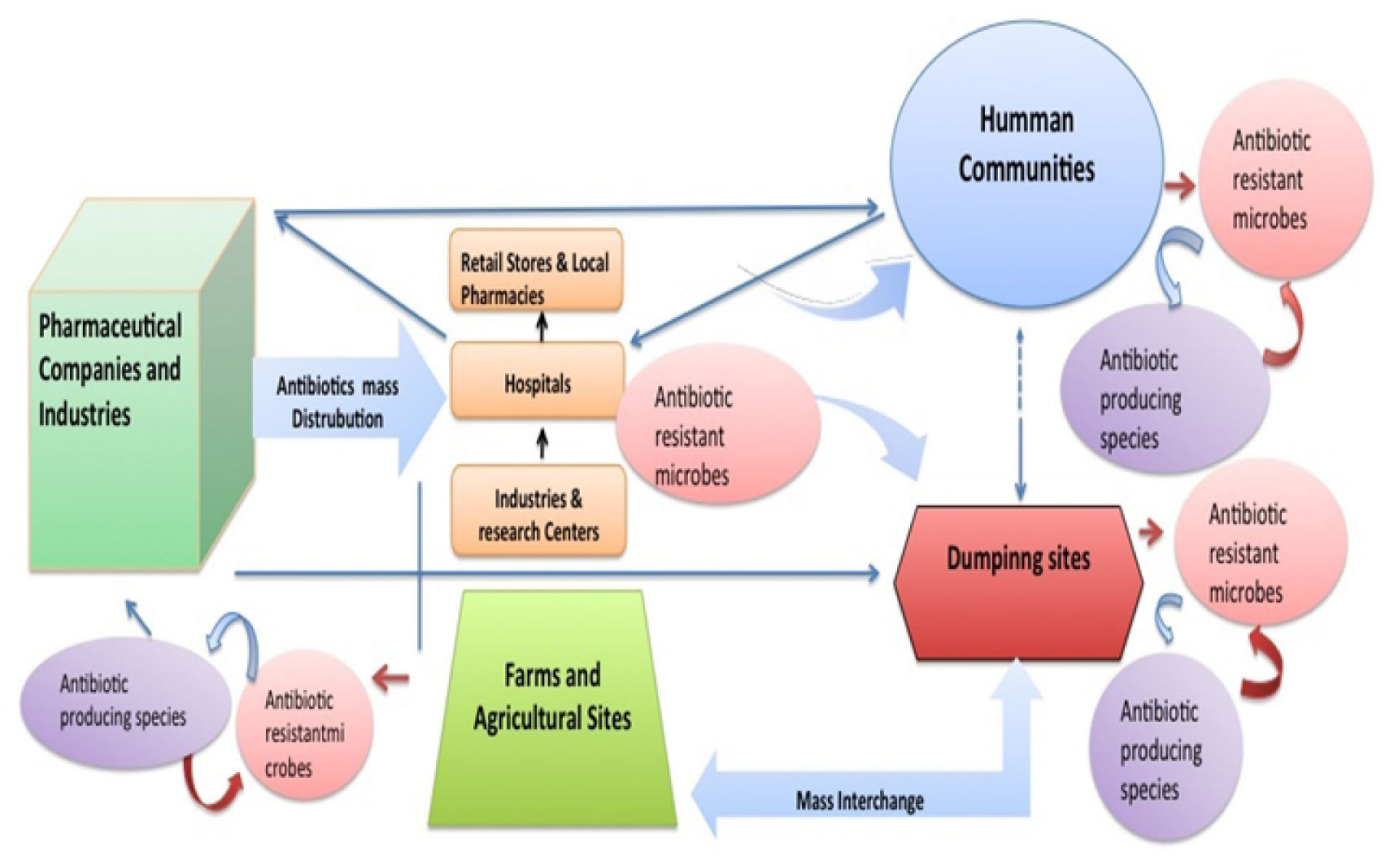
Schematic paradigm of antibiotic-producing microbiota upsurge and translocation in residential and recreational environments

The newly emerging cases of antibiotic-resistance have been emphatically linked to the increased use of antibiotics, pharmaceutical drugs, and antibactericidal soaps in human communities. Frequent usage of fertilizing agents in residential environments has also been shown to promote horizontal gene transfer, therefore expanding antibiotic-resistance in these locations [13,14]. Our investigation of antibiotic potentials within two distinct human populace niches *i.e.* ( residential and recreational sites) revealed a higher antibiotic-producing community in residential soil compared to recreational soil, supporting the notion of a growing antibiotic resistant microbiota leading to a parallel upsurge of antibiotic-producing microorganisms in heterogeneous microenvironments [15,16]. We’ve conceptualized a paradigm by which this microbial coevolution and interchange may take place between human populated niches and different environmental sites (Fig. 4).

Interestingly, most of the 16S rRNA-sequenced isolates (i.e. Y14p, Y16, and Y40p) have been previously associated with nosocomial infections [7,20,21]. This supports our hypothesis and may indicate that APS presence in residential soil is a consequence of translocational events, which likely brought them from a health care center to a residential location (Fig. 4), and their antibiotic-producing capacities to also be adaptive evolutionary acquisitions for survival in soil infiltrated with antibiotic-resistant strains. Further genomic analysis would be needed to validate the genotypic similarities of the APS isolates to that of their BLAST counterparts.

## 4. CONCLUSION

The abundance of antibiotic-producing microorganisms in residential soils compared to recreational soils supports our hypothesis. This is likely due in large to antibiotics overuse and the release of unused or un-metabolized antibiotics in residential environments [13,24], which likely leads to the rise of an extensively bioactive microbiota, thus diversifying and exponentially expanding the antibiotic-producing microbiota in those environments compared to recreational soils seldom exposed to antibiotic misuse or contaminations. It is also worth noting that the elevated levels of antibiotic-producing bacteria observed in residential soil may highlight a potential threat, as they may indicate the concurrent increase of unknown antibiotic resistant strains with robust shielding capacities beyond the inhibitory competence of contemporary medicinal antibiotics.

Our findings support previous studies demonstrating an increase in bioactive microbiota in places with high human activity, the limited number of sequenced soil isolates merely enables us to speculate on the possibility of a trend and does not permit us to confirm the frequency of antibiotic-producing microbial presence in residential microenvirons. Studies done on soils from various geographical locations would be needed to determine if this trend persists in most residential environments.

## Figures and Tables

**Table 1:** Count of residential and recreational soil isolates displaying antibiotic-producing capacities at different screening stages

		NA		PDA		TSA	

Screening stage	n	Res.	Rec.	Res.	Rec.	Res.	Rec.
Primary	80	52 (*73.2%)	9 (*100.0%)	12 (16.9%)	---	7 (9.9%)	---
Secondary	20	11 (*21.2%)	1 (*11.1%)	4 (*33.3%)	---	4 (*57.5%)	---
Agar-plug assay	15	8 (*88.9%)	1 (*100.0%)	2 (*100.0%)	---	4 (*100.0%)	---

%= population remaining from screening.

* P = .05

**Table 2:** Soil isolates displaying robust growth inhibitory properties after secondary screenings by cross-plate assay against 19 pathogens on three different media

ATCC Pathogens	Gram staining	PDA	NA	TSA
***B. cereus***	+	Y14, Y13	Y17, Y29, Y40, L59	Y64a
***L. monocytogenes***	+	---	Y10, Y49, Y16	Y66, Y64a, Y67, Y63, Y64b
***C. albicans***	Yeast	Y14	Y40, Y29, Y16a, Y17, L59	Y64
***E. coli***	−	Y13	---	---
***En. saccharolyticus***	+	---	Y8, Y10, Y21, Y23, Y40, Y17, Y29, Y39	Y64a, Y63, Y67, Y66
***En. faecalis***	+	---	---	Y63, Y64a
***K. kristiniae***	+	Y11, Y13, Y69, Y14	Y8, Y21, Y40, Y44, Y49, Y16, Y16a	Y67, Y63, Y64a
***K. pneumonia***	−	---	---	Y64, Y66
***K. oxytoca***	−	---	---	
***M. luteus***	+	Y13, Y14	Y23	Y63, Y64a
***O. anthropi***	−	Y11	Y8, Y40, Y49, Y16a	Y64b
***P. fluorescence***	−	Y13, Y14	Y17, Y29, Y40, L59	---
***S. cerevisiae***	Yeast	Y13, Y14	Y16, Y17	Y64a, Y63
***S. marcescess***	−	---	Y23	---
***S. aureus***	+	Y14	Y51, Y17, Y29, Y49, L59	Y67
***S. sciuri***	+	---	Y40, Y49, Y44, Y16, Y16a	Y64a, Y63, Y64b, Y67
***S. zooepidemicus***	+	---	Y17, Y29, Y40, L59	Y63
***S. pneumoniae***	+	---	Y40	Y64a, Y66
***S. thermophilus***	+	---	---	

Y= residential soil derived; L= recreational soil derived.

**Table 3:** Average ZOI (mm) displayed against 19 clinically relevant pathogens of APS-derived supernatant application on three different media

ATCC pathogens	PDA			NA								TSA			
	Y13	Y14^p^	Y16	Y16a	Y17^p^	Y29^p^	Y39	Y40^p^	Y44	Y49	L59	Y63^p^	Y64a	Y64b	Y67^p^
*B. cereus*	---	---	---	---	0.25	---	---	---	---	---	---	---	---	---	---
*L. monocytogenes*	---	1.02	2.54	2.41	---	---	---	---	---	---	---	2.54	1.52	5.08	2.54
*C. albicans*	---	2.03	---	1.14	2.54	---	---	---	---	---	2.54	---	2.54	---	---
*E. coli*	0.25	---	---	---	---	---	---	---	---	---	---	---	---	---	---
*En. saccharolyticus*	---	---	---	---	---	---	2.29	---	---	---	---	3.30	1.02	2.03	---
*En. faecalis*	---	---	---	---	---	---	---	---	---	---	---	0.25	---	---	---
*K. kristinae*	---	10.16	1.27	0.25	---	---	---	1.27	2.01	2.54	---	2.31	1.78	---	1.65
*K. pneumonia*	---	---	---	---	---	---	---	---	---	---	---	---	---	---	---
*K. oxytoca*	---	---	---	---	---	---	---	---	---	---	---	---	---	---	---
*M. luteus*	7.62	7.62	---	---	1.52	---	---	---	---	---	---	2.54	1.78	---	---
*O. anthropic*	---	---	---	2.54	---	---	---	---	---	---	---	1.27	---	1.40	---
*P. fluorescence*	0.25	3.94	---	---	2.54	2.54	---	2.54	---	---	2.54	---	---	---	---
*S. cerevisiae*	6.35	4.06	---	2.03	1.78	---	---	---	---	---	---	2.54	---	7.62	0.25
*S marcescess*	9.40	4.06	---	---	---	---	---	---	---	---	---	---	---	---	---
*S. aureus*	---	---	---	---	2.54	2.54	---	---	---	6.35	6.096	1.78	1.52	1.52	1.40
*S. sciuri*	---	---	0.89	1.78	---	---	---	1.02	---	---	---	0.76	1.78	2.03	1.52
*S. zooepiderimicus*	---	---	---	---	---	---	---	---	---	---	---	3.81	---	---	---
*S. pneumonia*	---	---	---	---	---	4.95	---	---	---	---	---	---	---	---	---
*S. thermophiles*	---	---	---	---	---	---	---	---	---	---	---	---	---	---	---

P= pDNA presence
